# Risk assessment of pulmonary infections in primary glomerular diseases treated with rituximab

**DOI:** 10.3389/fimmu.2025.1646894

**Published:** 2025-10-24

**Authors:** Chunyun Yang, Qiuyue Li

**Affiliations:** Department of Nephrology, The First Affiliated Hospital of Nanchang University, Nanchang, China

**Keywords:** rituximab, primary, glomerular diseases, pulmonary, infection

## Abstract

**Purpose:**

This study aimed to evaluate the incidence and risk factors of pulmonary infections in patients with primary glomerular diseases (PGDs) treated with rituximab (RTX).

**Methods:**

Clinical data of patients treated with RTX were collected, along with instances of pulmonary infections occurring within 6 months of RTX administration, to analyze risk factors associated with pulmonary infections in PGDs.

**Results:**

A total of 246 patients were included in the study, comprising 169 with idiopathic membranous nephropathy (IMN), 53 with minimal change disease (MCD), nine with focal segmental glomerulosclerosis (FSGS), and 15 with IgA nephropathy (IgAN). Within 6 months, 39 patients developed pulmonary infections, corresponding to an infection rate of 15.85%. The infected group had higher age, serum creatinine (Scr), white blood cells (WBC), neutrophils (NEUT), neutrophil percentage-to-albumin ratio (NPAR), and proportion of latent tuberculosis than the non-infected group (p < 0.05). Conversely, serum albumin (sALB), estimated glomerular filtration rate (eGFR), complement component 3 (C3), and hemoglobin (HGB) were lower in the infected group (p < 0.05). No significant differences were observed between the two groups concerning gender, body mass index (BMI), comorbidities (hypertension, diabetes, and hepatitis B), concomitant use of corticosteroids or immunosuppressants, total dose of RTX, blood pressure, lymphocytes (LYM), percentage of neutrophils (NEUT%), urine protein-to-creatinine ratio (UPCR), C-reactive protein (CRP), serum immunoglobulin G (sIgG), and B-cell counts (p > 0.05). Four feature variables were identified through Least absolute shrinkage and selection operator (LASSO) binary logistic regression: sALB, age, C3, and eGFR. Univariate Cox proportional hazards regression analysis included clinically relevant factors and those differing significantly between groups (p < 0.1), identifying latent tuberculosis (LTB), age, C3, and eGFR as potential risk factors for infection (p < 0.05). sALB was identified as a potential protective factor (p < 0.01). Multivariate Cox proportional hazards regression analysis indicated that sALB is an independent protective factor against infection (p < 0.05); age, C3, and eGFR are independent risk factors (p < 0.01). The restricted cubic splines (RCS) curves showed that the risk of infection increased with advancing age and higher sALB but decreased with eGFR and C3. Furthermore, a non-linear association was observed between RTX dose and infection risk.

**Conclusion:**

The incidence of pulmonary infections within 6 months of RTX treatment in patients with PGDs was 15.85%. sALB is a protective factor against pulmonary infections, while age, C3, and eGFR are risk factors. The total dose of RTX, concomitant use of corticosteroids or immunosuppressants, and levels of sIgG, LYM, NEUT, and NPAR were not related to infection risk.

## Introduction

Rituximab (RTX) is a chimeric monoclonal antibody engineered through recombinant DNA technology that specifically targets the transmembrane protein CD20 on B cells, binding to it and inducing cytotoxicity mediated by complement and antibodies, thus depleting peripheral B cells. The CD20 antigen is not expressed on hematopoietic stem cells, normal plasma cells, or other normal tissues, allowing RTX to selectively reduce B lymphocytes, inhibiting the production of autoantibodies without the non-specific immunosuppressive toxicity (1). As understanding of RTX has deepened, targeted therapy focusing on B cells has gained attention and is increasingly applied in clinical settings. The 2021 Kidney Disease Improving Global Outcomes (KDIGO) guidelines recommend RTX for treating various immune-mediated glomerular diseases, such as idiopathic membranous nephropathy (IMN), minimal change disease (MCD), and lupus nephritis (LN) (2). The consensus among experts in China also supports the use of RTX for various pathological types of glomerular nephritis, including IMN, MCD, focal segmental glomerulosclerosis (FSGS), Anti-Neutrophil Cytoplasmic Antibodies-associated vasculitis, and LN (3), and also in studies suggesting effectiveness in IgA nephropathy (IgAN) (4) and IgA vasculitis (5). In terms of safety, RTX is closely associated with severe infections, including *Pneumocystis jirovecii* pneumonia (PJP) and the reactivation of hepatitis B virus (HBV) and tuberculosis (TB). The mechanisms of infection risk involve B-cell depletion, B-cell–T-cell crosstalk, hypogammaglobulinemia, delayed neutropenia, and diminished immune response after vaccination (6). Studies have shown that RTX poses a significantly higher risk of infection in secondary membranous nephropathy (SMN) compared to primary glomerular diseases (PGDs) (7), particularly in older individuals, those with low baseline IgG levels, and a recent history of infections. In our center, RTX is more frequently used in PGDs; hence, this study aimed to explore the incidence and risk factors for pulmonary infections in PGDs treated with RTX to assist in clinical assessment and preventive measures.

## Materials and methods

### Patient population

Patients with PGDs treated with RTX at the First Affiliated Hospital Nephrology Department of Nanchang University from January 1, 2021, to February 1, 2024, were included in this study. The inclusion criteria were 1) age > 14 years and 2) PGDs confirmed by renal biopsy. The exclusion criteria were as follows: 1) secondary glomerulonephritis (SGN) due to autoimmune diseases, tumors, medications, or hematological conditions; 2) estimated glomerular filtration rate (eGFR) <30 mL/(min × 1.73 m^2^) or patients undergoing renal replacement therapy; 3) acute infections prior to RTX treatment; 4) incomplete clinical or laboratory data or lost to follow-up; 5) clinical remission achieved before RTX; 6) the use of other biologics within 6 months prior to RTX treatment; and 7) regular follow-up of <6 months.

### Clinical data collection

Data collected included gender, age, body mass index (BMI), baseline blood pressure, pathological type, hypertension, diabetes, urine protein-to-creatinine ratio (UPCR), serum albumin (sALB), serum creatinine (Scr), serum uric acid (SUA), alanine aminotransferase (ALT), aspartate aminotransferase (AST), C-reactive protein (CRP), triglycerides (TG), total cholesterol (TC), hemoglobin (HGB), white blood cells (WBC), platelets (PLT), lymphocytes (LYM), neutrophils (NEUT), neutrophil percentage (NEUT%), serum immunoglobulin G (sIgG), complement component 3 (C3), absolute B lymphocyte count, hepatitis B panel, T-cell spot test for tuberculosis infection (T-SPOT), and tuberculin skin test [purified protein derivative (PPD)]. Concomitant use of immunosuppressants, including glucocorticoids (GC), cyclophosphamide (CTX), tacrolimus (TAC), mycophenolate mofetil (MMF), and *Tripterygium wilfordii* polyglycoside tablet (TGT), was also noted. The eGFR was calculated using the Chronic Kidney Disease Epidemiology Collaboration (CKD-EPI) formula.

### Definition of infections and outcomes

Infections were graded according to the 2017 U.S. Department of Health and Human Services Common Terminology Criteria for Adverse Events (CTCAE) version 5.0. Patients with pulmonary infections with CTCAE 3 or above were included in this study for analysis. Patients with a positive T-SPOT and/or PPD prior to RTX were considered to have latent tuberculosis (8) and were treated with rifampin and isoniazid for 3 months or isoniazid alone for 6 months as a prophylactic measure. Rituximab was mixed with saline and administered after pre-medication with 5 mg of dexamethasone and 25 mg of promethazine by intramuscular injection to prevent acute infusion reactions. Treatment regimens included (9) the following: four-dose regimen, 375 mg/m^2^ per dose, weekly for 4 weeks; two-dose regimen, 1,000 mg per dose, every 2 weeks for a total of two doses; and B cell-guided protocol based on peripheral blood B-cell depletion. A small number of patients received intermittent low-dose therapy due to financial constraints, underlying comorbidities, or poor adherence. The total dose of RTX and concomitant immunosuppressant use (the initial GC dose was 0.5–0.6 mg·kg^−1^·day^−1^) were tapered by 50% every 2 weeks until discontinuation after response achievement. MMF was administered at 0.5g twice daily, TAC at 1 mg twice daily, CTX at 50 mg once daily, and TGT at two tablets three times daily. All medications were rapidly tapered and discontinued following achievement of clinical response. Pulmonary infection occurrence within 6 months was recorded. Patients were divided into infected and non-infected groups, and significant variables were included in univariate and multivariate logistic regression analyses to explore risk factors for RTX-associated pulmonary infections in PGDs.

### Ethics statement

The study was approved by the Ethics Committee of the First Affiliated Hospital of Nanchang University (IIT2024262) in accordance with the 1964 Declaration of Helsinki and amendments. Informed consent was not obtained from the participants, as it was a non-interventional retrospective data analysis of real-life data collected on patients’ regular visits.

### Statistical analysis

Data analysis was performed using R 4.3.2 and GraphPad Prism. Normally distributed continuous data were presented as mean ± standard deviation, non-normally distributed data as medians (P25–P75), and categorical data as frequencies (%). Comparisons between groups for normally distributed data were conducted using the t-test and for non-normally distributed data using the Wilcoxon rank-sum test; categorical data comparisons were conducted using the chi-square test; multiple group comparisons were conducted using the Kruskal–Wallis H test. Relationships between continuous variables and outcomes were described using restricted cubic splines (RCS). Cox proportional hazards regression was used to estimate the hazard ratio (HR) and 95% confidence interval (CI) for infection risk assessment. The proportional hazards assumption was verified using Schoenfeld residual tests. In the regression analysis model, covariates were selected based on clinical importance and statistical significance using the LASSO regression model. Multiple collinearity screening between covariates showed that all variance inflation factors (VIFs) were less than 5. A p-value <0.05 was considered statistically significant.

## Results

### Patient enrollment process

**Figure 1:**
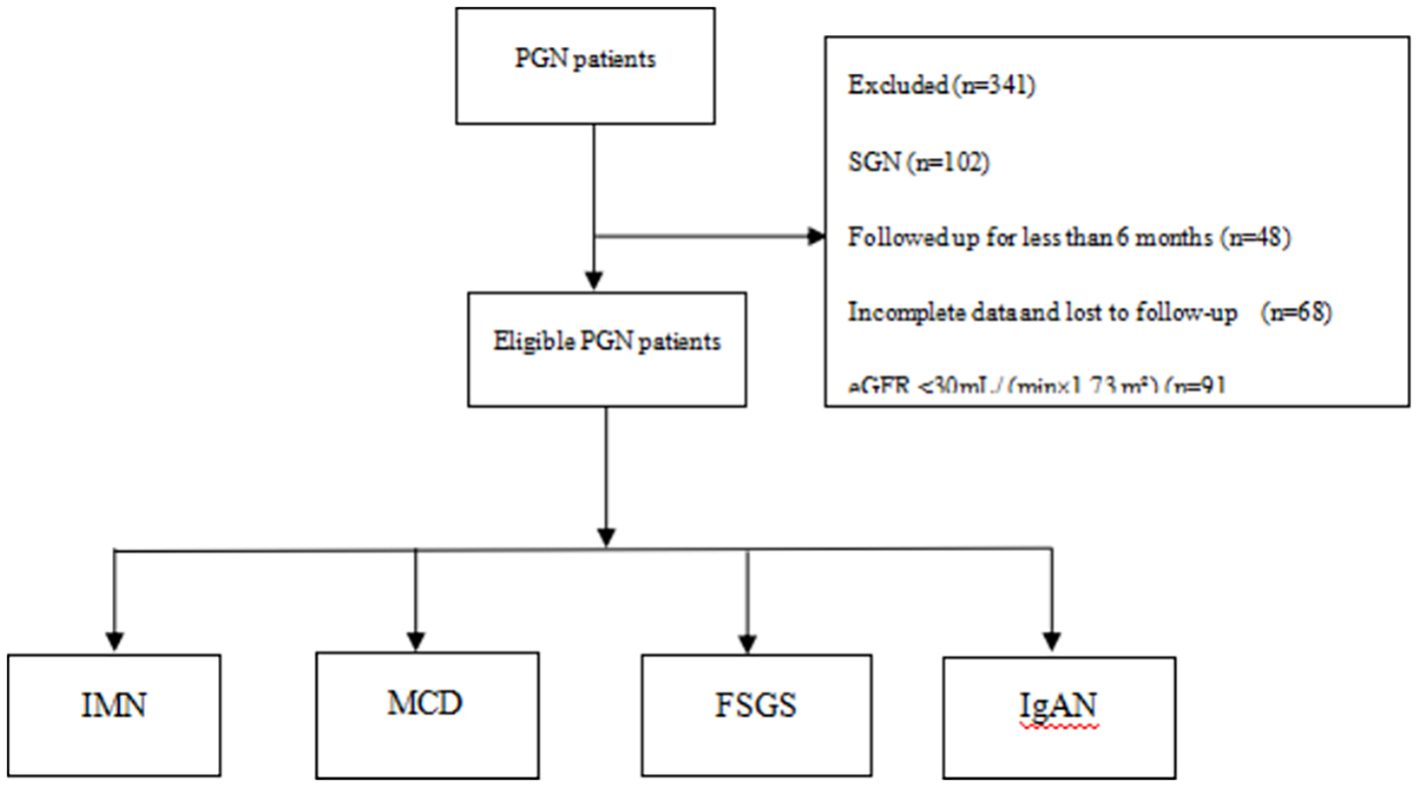
Flowchart for screening eligible patients.

A total of 587 patients were screened. Based on the inclusion and exclusion criteria, 246 patients were ultimately included in this study. The pathologies included IMN, MCD, FSGS, and IgAN; of these, 169 patients (68.7%) had IMN, 53 patients (21.5%) had MCD, nine patients (3.7%) had FSGS, and 15 patients (6.1%) had IgAN. The mean age of the patients was 48.0 years (interquartile range, 31.7 to 60.0), with 163 men (66.3%) and 83 women (33.7%). See **Figure 1** for details.

### Clinical data between the infected and non-infected groups

Of the 246 patients included, the median total dose of RTX was 2,000 mg, with 178 patients (72.4%) receiving a total RTX dose ≥2,000 mg and 68 patients (27.6%) receiving <2,000 mg. Of the patients, 62.6% were co-administered GC, 37.8% TAC, 4.9% CTX, 3.3% MMF, and 3.7% TGT. Within 6 months of follow-up, 39 patients (15.85%) developed pulmonary infections. Among these, infections occurred in 31 IMN patients (18.34%), five MCD patients (9.43%), one FSGS patient (11.11%), and two IgAN patients (13.33%). The median time to infection was 66.4 days (interquartile range, 37.5 to 93.5). Two patients died of respiratory failure, while the remaining patients were cured or achieved clinical improvement. Rituximab was discontinued in all cases following infection. Comparative analysis between the infected and non-infected groups revealed that the infected group had significantly higher age, Scr, WBC, NEUT, and proportion of latent tuberculosis (p < 0.05); in contrast, sALB, eGFR, C3, and HGB were lower (p < 0.05). There were no significant differences in gender, BMI, comorbidities (hypertension, diabetes, and hepatitis B), concurrent use of corticosteroids and immunosuppressants, total dose of RTX, blood pressure, ALT, AST, SUA, TC, TG, PLT, LYM, NEUT%, UPCR, CRP, IgG, and B lymphocyte counts (p > 0.05). See **Table 1** for details.

**Table 1:** Baseline data in infected and non-infected groups.

Clinical and laboratory data	Infected group (n = 39)	Non-infected group (n = 207)	t/z/χ^2^	P
IMN	31 (79.5)	138 (66.7)	2.73	0.435
MCD	5 (12.8)	48 (23.2)		
FSGS	1 (2.6)	8 (3.9)		
IgAN	2 (5.1)	13 (6.3)		
Male, n (%)	28 (71.8)	135 (65.2)	0.635	0.426
Female, n (%)	11 (28.2)	72 (34.8)		
Hypertension, n (%)	11 (28.2)	69 (33.3)	0.393	0.531
Diabetes, n (%)	4 (10.3)	31 (15.0)	0.599	0.439
Hepatitis B surface antigen positive, n (%)	6 (15.4)	30 (14.5)	0.021	0.885
Hepatitis B core antibody positive, n (%)	25 (64.1)	122 (58.9)	0.364	0.546
LTB, n (%)	6 (15.4)	12 (5.8)	4.448	0.035
Combined GC, n (%)	25 (64.1)	129 (62.3)	0.045	0.833
Combined CTX, n (%)	1 (2.6)	11 (5.3)	0.535	0.465
Combined TAC, n (%)	14 (35.9)	79 (38.2)	0.072	0.789
Combined MMF, n (%)	0 (0.0)	8 (3.9)	1.558	0.212
Combined TGT, n (%)	1 (2.6)	8 (3.9)	1.760	0.185
Grouping according to RTX dose, n (%)				
<2,000 mg	9 (23.1)	59 (28.5)	0.483	0.487
≥2,000 mg	30 (76.9)	148 (71.5)		
Total RTX dose (mg)	2,000 (2,000, 2,100)	2,000 (1,500, 2,000)	−1.493	0.135
Age	60.0 (40.0, 67.0)	47.0 (31.0, 58.0)	−3.406	0.001
BMI (kg/m^2^)	23.51 (20.94, 25.34)	24.22 (22.04, 26.67)	−1.560	0.119
Systolic blood pressure (mmHg)	134 (115, 144)	129 (115, 141)	−0.812	0.417
Diastolic blood pressure (mmHg)	81 (75, 90)	83 (77, 91)	−0.789	0.430
ALT (U/L)	17.2 (11.0, 24.3)	18.2 (13.0, 27.0)	−0.894	0.371
AST (U/L)	22.7 (14.0, 31.2)	21.3 (17.4, 27.8)	−0.044	0.965
sALB (g/L)	21.4 (18.1, 25.6)	24.4 (18.8, 32.5)	−2.156	0.031
Scr (μmol/L)	103.8 (71.0, 166.8)	78.5 (65.4, 108.2)	−2.799	0.005
eGFR (mL/min/1.73 m^2^)	63.86 (39.26, 96.40)	93.7 (67.92, 110.53)	−3.566	<0.001
SUA (μmol/L)	360.8 (294.3, 415.0)	380.3 (317.0, 452.8)	−1.423	0.155
TC (mmol/L)	7.04 (5.61, 9.31)	6.93 (5.38, 9.52)	−0.153	0.878
TG (mmol/L)	2.08 (1.71, 3.11)	2.41 (1.57, 4.02)	−1.072	0.284
WBC (10^9^/L)	8.86 (6.84, 10.71)	7.48 (5.81, 10.02)	−2.041	0.041
HGB (g/L)	122.9 ± 21.9	132.4 ± 22.55	−2.423	0.016
PLT (10^9^/L)	245 (185, 290)	252 (207, 309)	−0.347	0.728
LYM (10^9^/L)	1.37 (1.08, 2.36)	1.56 (1.19, 2.07)	−0.722	0.470
NEUT (10^9^/L)	6.38 (3.95, 8.35)	4.65 (3.51, 7.56)	−2.068	0.039
NEUT%	67.60 (61.80, 79.60)	67.60 (59.5, 75.5)	−0.747	0.455
NPAR	3.18 (2.50, 3.89)	2.57 (2.03, 3.74)	−2.530	0.011
CRP (mg/L)	2.15 (0.80, 9.54)	1.08 (0.80, 2.78)	−1.650	0.099
IgG (g/L)	5.72 (3.26, 8.07)	5.85 (3.75, 8.52)	−0.370	0.712
C3 (g/L)	0.85 ± 0.17	0.94 ± 0.21	2.666	0.008
B-cell count	209.0 (120.0, 371.0)	206.0 (107, 340)	−0.587	0.557
UPCR (g/g)	5.35 (3.47, 11.09)	5.37 (2.98, 9.02)	−1.152	0.249

IMN, idiopathic membranous nephropathy; MCD, minimal change disease; FSGS, focal segmental glomerulosclerosis; IgAN, IgA nephropathy; LTB, latent tuberculosis; GC, glucocorticoids; CTX, cyclophosphamide; TAC, tacrolimus; MMF, mycophenolate mofetil; TGT, *Tripterygium wilfordii* polyglycoside tablet; RTX, rituximab; BMI, body mass index; ALT, alanine aminotransferase; AST, aspartate aminotransferase; sALB, serum albumin; Scr, serum creatinine; eGFR, estimated glomerular filtration rate; SUA, serum uric acid; TC, total cholesterol; TG, triglycerides; WBC, white blood cells; HGB, hemoglobin; PLT, platelets; LYM, lymphocytes; NEUT, neutrophils; NEUT%, percentage of neutrophils; NPAR, neutrophil percentage-to-albumin ratio; CRP, C-reactive protein; IgG, immunoglobulin G; C3, complement component 3; UPCR, urine protein-to-creatinine ratio.

### Variable selection using LASSO regression

**Figure 2:**
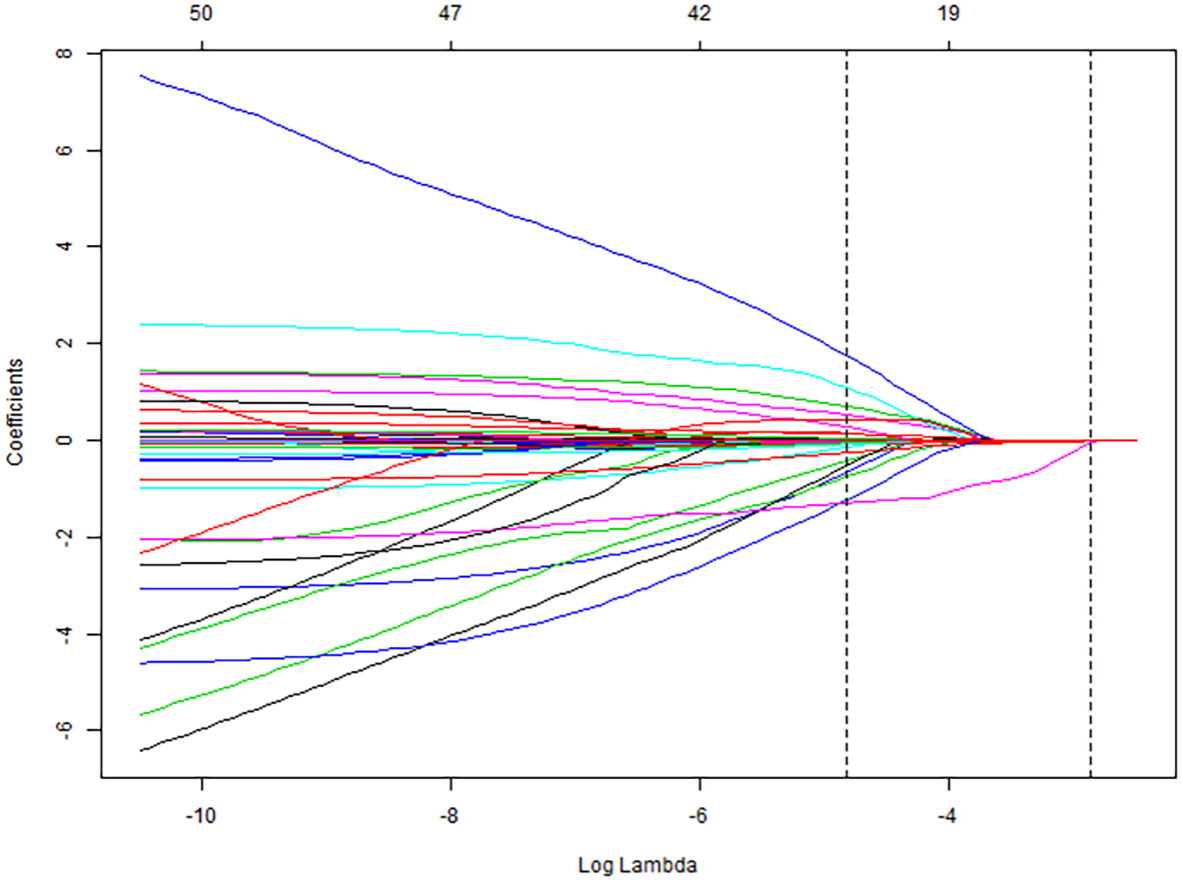
LASSO regression model coefficient paths.

**Figure 3:**
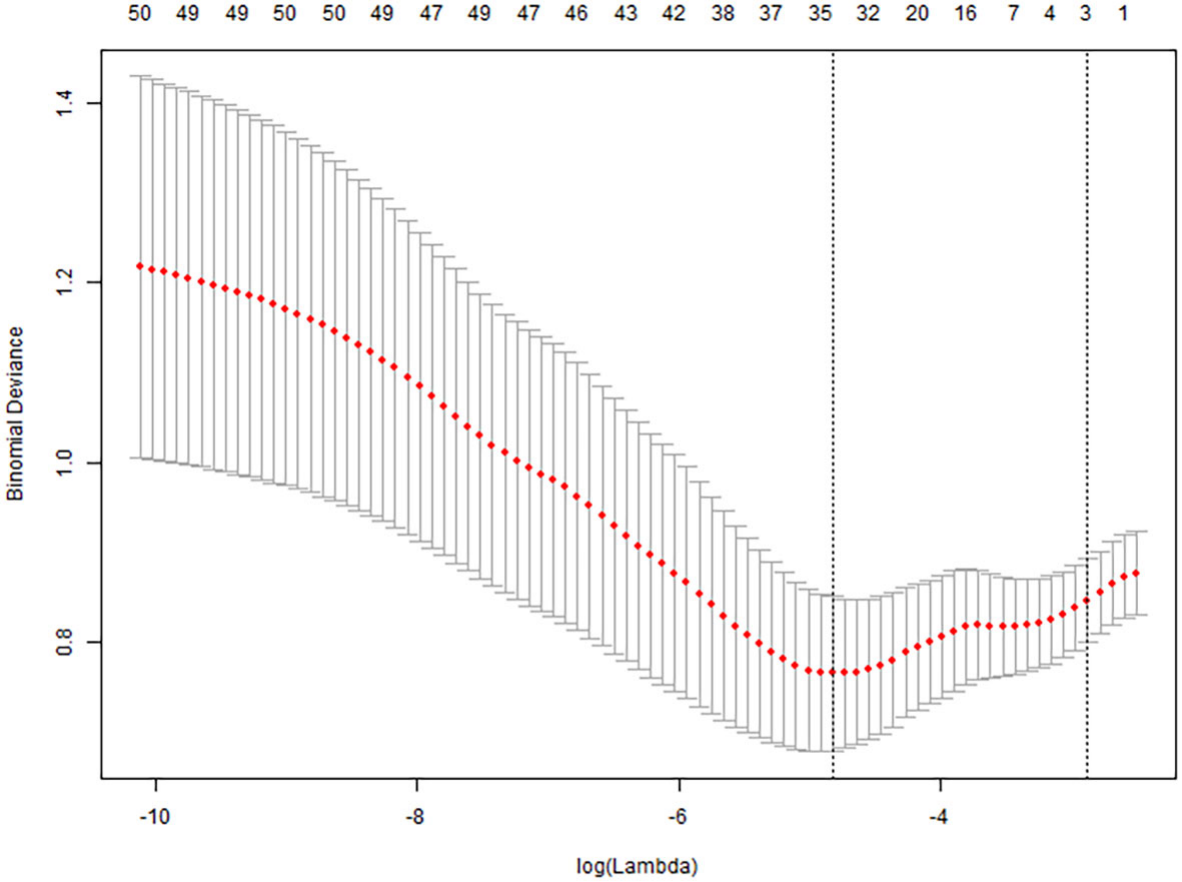
Cross-validation error of LASSO regression.

All collected clinical data were incorporated into a LASSO regression model, with the occurrence of pulmonary infection as the dependent variable. The LASSO binary logistic regression analysis identified predictors with non-zero coefficients, ultimately yielding four feature variables—sALB, age, C3, and eGFR—as detailed in **Figures 2** and **3**.

### Univariate and multivariate analyses of pulmonary infection outcomes

Univariate Cox proportional hazards regression analysis included clinically relevant factors and those differing significantly between groups (p < 0.1), identifying latent tuberculosis (LTB), age, C3, and eGFR as potential risk factors for infection (p < 0.05). sALB was identified as a potential protective factor (p < 0.01). Multivariate Cox proportional hazards regression analysis, incorporating clinical indicators significant in univariate analysis (p < 0.1), demonstrated that sALB is an independent protective factor against infection (p < 0.05). Age, C3, and eGFR are independent risk factors for infection (p < 0.01). Detailed results are shown in **Table 2**.

**Table 2:** Univariate and multivariate Cox proportional hazards regression analyses of infection outcomes.

Clinical and laboratory data	Univariate Cox analysis		Multivariate Cox analysis	
	HR (95% CI)	P	HR (95% CI)	P
LTB	2.45 (1.02−5.89)	0.045		
Age	1.02 (1.00−1.04)	0.024	1.02 (1.00−1.04)	0.025
BMI	0.94 (0.87−1.01)	0.110		
sALB	1.025 (1.01−1.04)	0.009	1.019 (1.001−1.038)	0.035
Scr	1.00 (1.00−1.00)	0.179		
eGFR	0.38 (0.51−0.90)	0.008	0.67 (0.50−0.90)	0.007
WBC	1.01 (0.94−1.10)	0.732		
HGB	0.99 (0.97−1.00)	0.053		
PLT	1.00 (1.00−1.00)	0.652		
NEUT	1.03 (0.94−1.12)	0.550		
NEUT%	1.01 (0.99−1.03)	0.493		
IgG	0.99 (0.87−1.13)	0.882		
B-cell count	1.00 (1.00−1.00)	0.700		
NPAR	1.11 (0.87−1.42)	0.111		
C3	0.12 (0.02−0.65)	0.014	0.14 (0.02−0.79)	0.026

LTB, latent tuberculosis; BMI, body mass index; sALB, serum albumin; Scr, serum creatinine; eGFR, estimated glomerular filtration rate; WBC, white blood cells; HGB, hemoglobin; PLT, platelets; NEUT, neutrophils; NEUT%, percentage of neutrophils; IgG, immunoglobulin G; NPAR, neutrophil percentage-to-albumin ratio; C3, complement component 3.

### Saturation effect of age, eGFR, C3, sALB, and RTX on pulmonary infection

**Figure 4:**
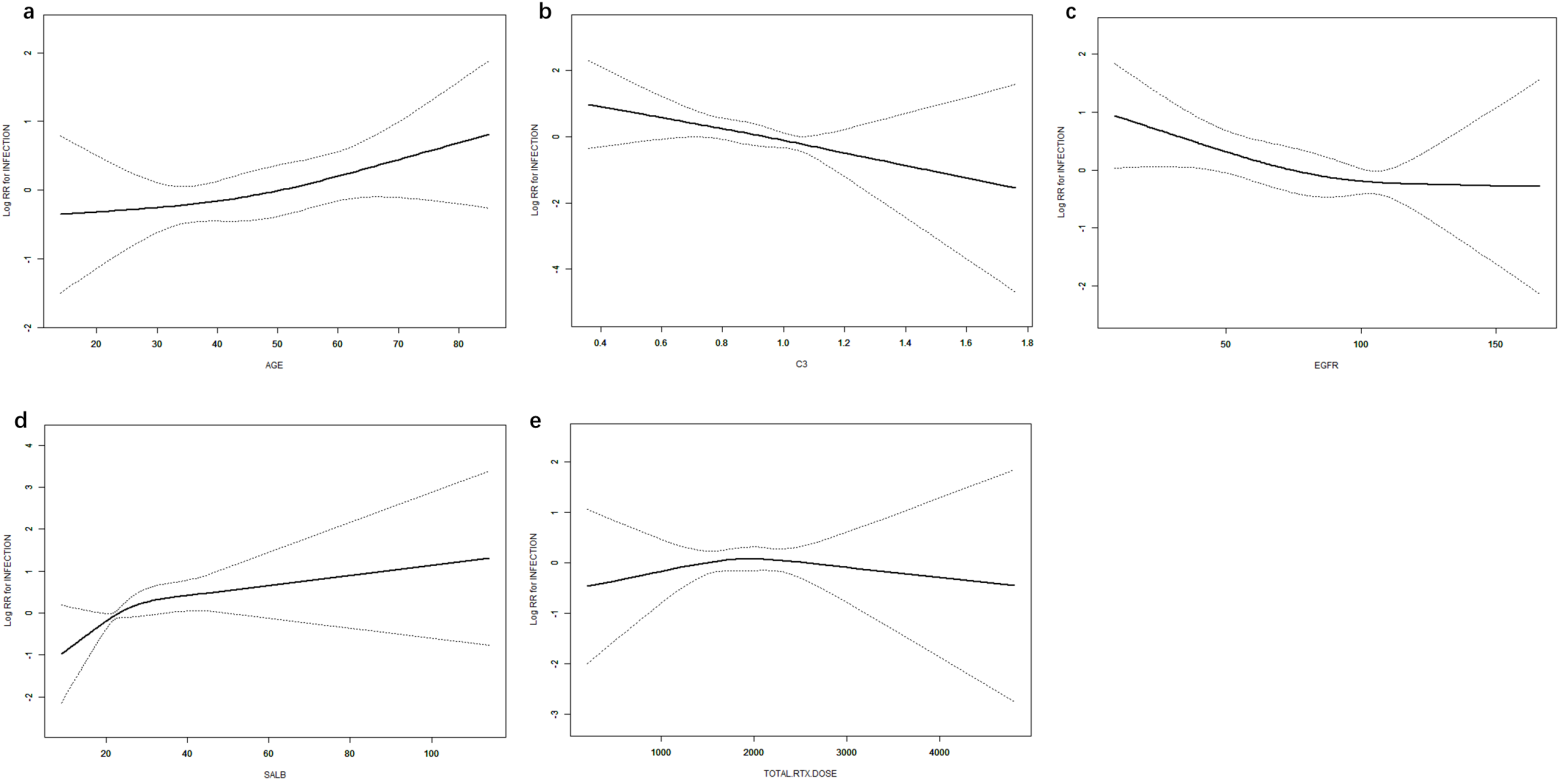
Dose–response relationships between age **(a)**, C3 **(b)**, eGFR **(c)**, ALB **(d)**, RTX **(e)**, and infection. Models were adjusted for sex, age, BMI, SBP, DBP, pathological type, sALB, Scr, eGFR, WBC, HGB, PLT, NEUT, NEUT%, IgG, B-cell count, CRP, and NPAR. C3, complement component 3; eGFR, estimated glomerular filtration rate; ALB, albumin; RTX, rituximab; BMI, body mass index; SBP, systolic blood pressure; DBP, diastolic blood pressure; sALB, serum albumin; Scr, serum creatinine; HGB, hemoglobin; PLT, platelets; NEUT, neutrophils; NEUT%, percentage of neutrophils; IgG, immunoglobulin G; CRP, C-reactive protein; NPAR, neutrophil percentage-to-albumin ratio.

Age, eGFR, and C3 were used as continuous variables, and RCS were used to fit smooth curves. As shown in **Figure 4**, the risk of infection increased with higher age and sALB levels, decreased with increasing eGFR and C3 levels, and showed a non-linear pattern with RTX—rising initially and then declining. Threshold effect analysis indicated a significant threshold for ALB (p < 0.05), with the estimated inflection point (95% CI) at 14.7 g/L (14.7–17.1 g/L) based on the log-likelihood ratio test (p < 0.001). No significant threshold effects were observed for the other four variables (p > 0.05) (**Table 3**).

**Table 3:** Saturation effect of age, eGFR, C3, sALB, and RTX on pulmonary infection.

Pulmonary infection	HR, 95% CI, p-value				
	Age	eGFR	C3	sALB	RTX
Model I					
Standard linear model	1.02 (0.99, 1.04) 0.1366	0.99 (0.98, 1.00) 0.0453	0.17 (0.03, 1.02) 0.0523	1.02 (1.00, 1.04) 0.0257	1.00 (1.00, 1.00) 0.8913
Model II					
Threshold (K)	70	28.09	0.72	14.7	2,500
95% CI for K	63, 73	26.39, 50.57	0.66, 0.76	14.7, 17.1	2,100, 2,700
<K	1.02 (1.00, 1.05) 0.0732	1.03 (0.94, 1.14) 0.5079	28.05 (0.01, 145,151.29) 0.4448	Inf. (0.00, Inf.) 0.9929	1.00 (1.00, 1.00) 0.1992
>K	0.93 (0.77, 1.12) 0.4335	0.99 (0.98, 1.00) 0.0282	0.06 (0.00, 0.69) 0.0241	1.02 (1.00, 1.04) 0.1094	1.00 (1.00, 1.00) 0.1987
Log-likelihood ratio test	0.27	0.352	0.147	<0.001	0.08

Model I assumed a linear relationship to estimate the overall effect size between each exposure variable and the outcome (infection).

Model II incorporated non-linear relationships using restricted cubic splines to identify potential inflection points and to estimate the effect sizes within each segment.

The outcome variable was infection. Exposure variables included age, eGFR, C3, sALB, and total RTX dose. Covariates adjusted for in the models were BMI, gender, pathology type, systolic blood pressure (SBP), diastolic blood pressure (DBP), and NPAR.

eGFR, estimated glomerular filtration rate; C3, complement component 3; sALB, serum albumin; RTX, rituximab; BMI, body mass index; NPAR, neutrophil percentage-to-albumin ratio.

## Discussion

With the increasing use of biologics in immunologically mediated glomerular diseases, their safety in terms of infection risk has become increasingly critical. The RI-CYCLO study posited that there is no difference in efficacy and safety between rituximab and a cyclical regimen of GC and CTX in treating IMN (10). A cohort study of 135 IMN patients with a median age of 67 years followed up for 4 years demonstrated that age is a reliable predictor of infection risk (11), likely due to immunosenescence associated with aging, which impairs resistance to infections (12). In patients over 70 years old treated with RTX, while a 70% complete remission rate could still be achieved, the rate of fatal pulmonary infections was 8.1% (13). Our study in the PGD population similarly confirmed that advanced age is a risk factor for pulmonary infections.

The Trivin study (14) identified diabetes and cumulative RTX dose as independent risk factors for infections among patients with various types of glomerulopathies. Even among 90% of patients maintaining normal sIgG levels, a higher cumulative dose of RTX was associated with increased infection risk (15). However, this phenomenon was not observed in our study, possibly because the target population was PGD patients, and those with diabetes were relatively few, only 14.2%. Grouping by an RTX dose threshold of 2,000 mg did not reveal any impact of cumulative RTX dose on infection risk, possibly because all patients underwent thorough infection risk assessments before RTX therapy, allowing adequate dosing of 2,000 mg RTX in low-risk patients, whereas high-risk patients, including those of advanced age, with baseline pulmonary disease, or with low lymphocyte counts, received intermittent low doses based on B-cell depletion. The study of Odler et al. (16) suggested that factors closely associated with infection occurrence included low BMI and high baseline Scr, and GC treatment concurrently. Despite a 58.5% incidence of hypogammaglobulinemia, it was not associated with infection risk.

In our study, infections resolved after the reduction or discontinuation of immunosuppressants and antibiotic treatment, except for one patient who died from respiratory failure after being transferred to the Intensive Care Unit (ICU), and one patient discontinued treatment and was discharged against medical advice.

In addition to pulmonary infections, rare cases of other infections were not included in our study, such as herpes zoster, gastrointestinal infections, and cellulitis.

Our analysis identified sALB as an independent protective factor. Complete innate and adaptive immune responses depend on sALB; oxidative degradation of sALB can impact interactions with bioactive lipid mediators crucial for antimicrobial defense and repair, leading to impaired immune function, pulmonary edema, and fluid retention, which in turn promote infection development (17). Hypoalbuminemia is also an independent prognostic factor for various cardiovascular diseases (18, 19). Hypoalbuminemia is associated with the acquisition and severity of infectious diseases. There is bio-mechanistic plausibility for a causal link between hypoalbuminemia and increased risks of primary and secondary infections. Serum albumin levels have a prognostic value for complications in viral, bacterial, and fungal infections. Hypoalbuminemia predicts the development of healthcare-associated infections, particularly with *Clostridium difficile* (17).

Neutrophil percentage-to-albumin ratio (NPAR) emerged as a new predictive marker for poor infection prognosis, which proved to be more accurate than albumin or neutrophil percentage alone (20). NPAR, as a novel biomarker for systemic inflammation and infection, is receiving increasing attention. Studies have identified the optimal threshold of NPAR for predicting stroke-related infection incidence as 1.64 (21). Various studies have demonstrated that NPAR can serve as a prognostic indicator for patients with cardiogenic shock (22), myocardial infarction (23), acute kidney injury (24), and pancreatic cancer (25). In our study, the infection group had significantly higher NPAR values than the non-infected group, but further large-scale clinical studies are needed to confirm this as a risk factor.

Our study included 68.7% of patients with IMN, which is the most common primary renal syndrome in middle-aged and elderly patients, characterized by a higher incidence of hypertension, more severe glomerulosclerosis and interstitial fibrosis, and lower eGFR, leading to a higher risk of progressing to end-stage renal disease (ESRD) and higher infection risk, especially under immunosuppressive treatment (13). Multiple studies have shown that baseline creatinine is a high-risk factor for infection (16, 26), aligning with our findings.

In autoimmune responses, underlying infections are autoimmunity against three immunological targets: neutrophils, complement, and cytokines. The complement system is involved in both innate and adaptive immunity. It comprises three major pathways: the classical pathway (the activation of which is dependent on antibodies) and the lectin and alternative pathways (which can be directly activated by microorganisms, without the involvement of adaptive immunity) (27). Renal diseases are frequently mediated by immune complexes via the classical pathway, whose activation is antibody-dependent. While the pathogenic role of autoantibodies against complement molecules is often unclear, specific autoantibodies targeting the C3 convertase can enhance its activity and reduce complement levels, thereby predisposing individuals to severe bacterial infections. Our study demonstrated that decreased C3 is an independent risk factor for pulmonary infections following RTX treatment.

Pulmonary infections, which progress rapidly and can easily lead to respiratory failure, lack specific clinical and CT features, sometimes requiring Next-Generation Sequencing (NGS) of bronchoalveolar lavage fluid for definitive diagnosis, highlighting the importance of preventing pulmonary infections. Therefore, it is advised that special attention and careful risk assessment be given to patients over 50, especially those with diabetes, chronic pulmonary diseases, malnutrition, low serum IgG, and renal impairment, possibly administering Sulfamethoxazole prophylactically. Thus, individualized prevention and treatment strategies, including screening for tuberculosis, are of critical importance. Recommendations from the National Clinical Research Center for Infectious Diseases (28) suggest routine prophylactic antituberculosis treatment for 3 to 9 months for patients requiring long-term use of immunosuppressants with LTB. Prophylactic regimens included isoniazid monotherapy (300 mg once daily for 6 or 9 months), isoniazid plus rifapentine (once weekly for 3 months), isoniazid plus rifampin (once daily for 3 months), isoniazid plus rifapentine (once daily for 1 month), and rifampin monotherapy (600 mg once daily for 4 months). In adaptive immune responses, immunity against tuberculosis is primarily mediated by T cells. Prior to the initiation of a CD20 antagonist, neither LTB screening nor prophylactic antituberculosis treatment is required. However, prophylactic treatment for LTB is recommended in patients receiving GC at doses exceeding 15 mg, CTX, MMF, or TAC. No cases of TB infection were observed among all pulmonary infections reported in our study.

The lack of effect of concomitant immunosuppressant use on pulmonary infection outcomes could be influenced by underlying diseases, economic conditions, and compliance, among other factors, making it difficult to standardize treatment protocols. The variability in RTX dosing and duration of use among study participants, as well as potential recall bias, may have influenced these results. The sample sizes for FSGS and IgAN were relatively limited. In these patient populations, RTX was administered only after rigorous pathological selection. For instance, in IgAN, RTX was considered only when the rate of foot process effacement exceeded 70%. In FSGS, pathological changes were minimal and more consistent with those in MCD. Both groups consisted predominantly of young patients and exhibited a relatively low incidence of infection. Future studies should aim to expand the sample size within these pathological subtypes.

In conclusion, the study primarily explored the occurrence and influencing factors of pulmonary infections in PGD patients treated with RTX, providing a theoretical basis and clinical experience for the treatment and prevention of pulmonary infections. The study has certain limitations, including its single-center, retrospective design, a relatively short follow-up duration, and a lack of external validation. Therefore, further confirmation through subsequent multicenter prospective studies is warranted.

## Data Availability

The raw data supporting the conclusions of this article will be made available by the authors, without undue reservation.

## Glossary

PGDs: primary glomerular diseases
RTX: rituximab
IMN: idiopathic membranous nephropathy
MCD: minimal change disease
FSGS: focal segmental glomerulosclerosis
IgAN: IgA nephropathy
Scr: serum creatinine
WBC: white blood cells
NEUT: neutrophils
NPAR: neutrophil percentage-to-albumin ratio
sALB: serum albumin
eGFR: estimated glomerular filtration rate
HGB: hemoglobin
BMI: body mass index
LYM: lymphocytes
UPCR: urine protein-to-creatinine ratio
CRP: C-reactive protein
sIgG: serum immunoglobulin G
C3: complement component 3
LN: lupus nephritis
PJP: *Pneumocystis jirovecii* pneumonia
HBV: hepatitis B virus
LTB: latent tuberculosis
SMN: secondary membranous nephropathy
SUA: serum uric acid
ALT: alanine aminotransferase
AST: aspartate aminotransferase
TG: triglycerides
TC: total cholesterol
PLT: platelets
T-SPOT: T-cell spot test for tuberculosis infection
PPD: purified protein derivative
GC: glucocorticoids
CTX: cyclophosphamide
TAC: tacrolimus
MMF: mycophenolate mofetil
TGT: *Tripterygium wilfordii* polyglycoside tablet
